# Association between dietary patterns and cognitive function among 70-year-old Japanese elderly: a cross-sectional analysis of the SONIC study

**DOI:** 10.1186/s12937-017-0273-2

**Published:** 2017-09-11

**Authors:** Hitomi Okubo, Hiroki Inagaki, Yasuyuki Gondo, Kei Kamide, Kazunori Ikebe, Yukie Masui, Yasumichi Arai, Tatsuro Ishizaki, Satoshi Sasaki, Takeshi Nakagawa, Mai Kabayama, Ken Sugimoto, Hiromi Rakugi, Yoshinobu Maeda, SONIC Study Group, Madoka Ogawa, Yoshiko Lily Ishioka, Chisato Inomata, Hirochika Ryuno, Ryosuke Oguro, Koichi Yamamoto, Yasushi Takeya, Yoichi Takami, Norihisa Ito

**Affiliations:** 1 Department of Health Promotion, National Institute of Public Health, 2-3-6 Minami, Wako-shi, Saitama, 351-0197 Japan; 2 Tokyo Metropolitan Geriatric Hospital and Institute of Gerontology, Tokyo, Japan; 3 Department of Clinical Thanatology and Geriatric Behavioral Science, Osaka University Graduate School of Human Sciences, Osaka, Japan; 4 Department of Health Science, Osaka University Graduate School of Medicine, Osaka, Japan; 5 Department of Prosthodontics, Gerontology and Oral Rehabilitation, Osaka University Graduate School of Dentistry, Osaka, Japan; 6 Center for Supercentenarian Medical Research, Keio University, School of Medicine, Tokyo, Japan; 7 Department of Social and Preventive Epidemiology, School of Public Health, The University of Tokyo, Tokyo, Japan; 8 University Research Priority Program “Dynamics in Healthy Aging”, The University of Zurich, Zurich, Switzerland and Japan Society for the Promotion of Science, Tokyo, Japan; 9 Department of Geriatric and General Medicine, Osaka University Graduate School of Medicine, Osaka, Japan

**Keywords:** Cognitive function, Dietary pattern, Factor analysis, Japanese, Elderly

## Abstract

**Background:**

An increasing number of studies in Western countries have shown that healthy dietary patterns may have a protective effect against cognitive decline and dementia. However, information on this relationship among non-Western populations with different cultural settings is extremely limited. We aim to examine the relationship between dietary patterns and cognitive function among older Japanese people.

**Methods:**

This cross-sectional study included 635 community-dwelling people aged 69–71 years who participated in the prospective cohort study titled Septuagenarians, Octogenarians, Nonagenarians Investigation with Centenarians (SONIC). Diet was assessed over a one-month period with a validated, brief-type, self-administered diet history questionnaire. Dietary patterns from thirty-three predefined food groups [energy-adjusted food (g/d)] were extracted by factor analysis. Cognitive function was assessed using the Japanese version of the Montreal Cognitive Assessment (MoCA-J). Multivariate regression analysis was performed to examine the relationship between dietary patterns and cognitive function.

**Results:**

Three dietary patterns were identified: the ‘Plant foods and fish’, ‘Rice and miso soup’, and ‘Animal food’ patterns. The ‘Plant foods and fish’ pattern, characterized by high intakes of green and other vegetables, soy products, seaweeds, mushrooms, potatoes, fruit, fish, and green tea, was significantly associated with a higher MoCA-J score [MoCA-J score per one-quartile increase in dietary pattern: β = 0.56 (95% CI: 0.33, 0.79), *P* for trend <0.001]. This association was still evident after adjustment for potential confounding factors [β = 0.41 (95% CI: 0.17, 0.65), *P* for trend <0.001]. In contrast, neither the ‘Rice and miso soup’ nor the ‘Animal food’ pattern was related to cognitive function. To confirm the possibility of reverse causation we also conducted a sensitivity analysis excluding 186 subjects who reported substantial changes in their diet for any reason, but the results did not change materially.

**Conclusion:**

This preliminary cross-sectional study suggests that a diet with high intakes of vegetables, soy products, fruit, and fish may have a beneficial effect on cognitive function in older Japanese people. Further prospective studies are needed to confirm this finding.

**Electronic supplementary material:**

The online version of this article (doi:10.1186/s12937-017-0273-2) contains supplementary material, which is available to authorized users.

## Background

With a global aging population, the prevalence of age-related cognitive decline leading to dementia is rising dramatically, and is now one of the most serious public health challenges worldwide [1]. As there is still no effective cure or treatment to modify the course of dementia, successful preventive strategies will rely on accurate understanding of the role of modifiable factors and their influence on cognitive function and the risk of dementia.

There is considerable interest in the role of diet as an important modifiable risk factor for early prevention and delayed onset of age-related cognitive decline and dementia [2–6]. Much of the attention regarding the relation of diet to cognition or dementia among the elderly has shifted from the roles of single nutrients or foods to the roles of dietary patterns, reflecting the complexity of diet and our daily eating behavior. Recent systematic reviews and meta-analyses have summarized the available studies on the roles of dietary patterns in cognitive function and the risk of dementia [2–7]. Although several approaches to the identification of dietary patterns have been explored, the results in general suggest that dietary patterns with a higher intake of fruits, vegetables, fish, nuts, and legumes and a lower intake of meat and high-fat dairy products seem to be associated with better cognitive function or reduced risk of dementia [2–7]. In particular, the Mediterranean dietary pattern has shown, with considerable consistency, that it may protect against or slow the rate of cognitive decline with aging [3–5, 7]. Almost all of these studies, however, have been conducted in Western populations, while research in non-Western populations, including the Japanese population, is extremely limited [8–12]. The Japanese diet has characteristics seldom observed in those of Western populations, including high intakes of rice, soybean products, fish, seaweeds, and green tea, in addition to low total and saturated fat intakes [13]. Given the differences in dietary habits and social characteristics between the Japanese and Western cultures, further studies are warranted to examine the relationships between dietary patterns and cognitive function in the Japanese population.

The objective of the present cross-sectional study was to examine the association between dietary patterns and cognitive function among 635 older Japanese people who participated in a prospective cohort study called Septuagenarians, Octogenarians, Nonagenarians Investigation with Centenarians (SONIC) [14]. We considered the effects of a wide range of potential confounding factors, including sociodemographic characteristics, lifestyle, medical conditions, and genetic polymorphisms that have been suggested to influence cognitive function.

## Methods

### Study population and procedure

The present cross-sectional analysis was based on the baseline survey of the SONIC Study, an ongoing prospective cohort study of older people as part of the Centenarian Study that was initiated in June 2010 [14]. The principal objectives of the SONIC Study are to assess the characteristics of a general population sample of older people consisting of three control groups of different ages (70s, 80s, and 90s) for a centenarian cohort, and to clarify the factors for health, longevity, and psychological well-being. We used a narrow age-range cohort design [14], and each cohort includes individuals whose ages fall within a 3-year range (i.e., 69–71, 79–81, and 89–91 years old for the 70s, 80s, and 90s cohorts, respectively). The data collection for each age cohort was conducted in different years. The first-wave survey began in 2010 for the 70s cohort, 2011 for the 80s cohort, and 2012 for the 90s cohort. We have also conducted a follow-up assessment on each cohort every 3 years after the baseline survey. Each cohort consisted of a total of 1000 participants living independently who had been randomly selected through the local resident registry from four areas in Japan: Itami City, Hyogo (Western-urban); Asago City, Hyogo (Western-rural); Itabashi Ward, Tokyo (Eastern-urban); and Nishitama County, Tokyo (Eastern-rural). A detailed description of the rationale, study design, and protocol has been published elsewhere [14–16].

The present analysis includes only participants in the baseline survey of the 70s cohort. The baseline assessment of the SONIC Study was primarily conducted by means of a questionnaire booklet containing questions on socioeconomic status, psychosocial variables, medical conditions of self and family, dental conditions, diet, and lifestyle; medical and physical examinations were also performed. Research technicians confirmed missing or illogical data through interview at the survey venue. All measurements at each survey venue were conducted according to the survey protocol. A total of 1000 subjects (479 men and 521 women) aged 69–71 years completed the baseline survey for the 70s cohort between June and November 2010. After we excluded subjects who were currently receiving dietary counselling from a doctor or dietician (*n* = 123) or were missing information on the variables used (*n* = 242), data on 635 subjects (292 men and 343 women) were used for the present analysis.

### Dietary assessment

Dietary habits during the previous month were assessed using a brief-type self-administered diet history questionnaire (BDHQ) [17, 18]. The BDHQ for older people is a 10-page structured questionnaire inquiring about the consumption frequency of selected foods commonly consumed in Japan, general dietary behavior and usual cooking methods. Daily intake of foods (58 food items in total), energy, and selected nutrients was calculated using an ad hoc computer algorithm for the BDHQ, which was based on the Standard Tables of Food Composition in Japan [19]. Information on portion size was not collected in the BDHQ; rather, a sex-specific fixed portion size was used in calculations for all subjects. The relative validity of the estimates of intake from the BDHQ was established in comparison with intakes assessed using 16-day semi-weighed dietary records (DR) in a validation study of 92 Japanese men aged 32–76 years and 92 Japanese women aged 31–69 years. Median Spearman correlation coefficients with the DR for the 58 foods were 0.48 (range: 0.22–0.83) in men and 0.44 (range: 0.14–0.82) in women [17]. Median Pearson correlation coefficients with the DR for energy-adjusted intakes of the 42 nutrients were 0.56 (range: 0.41–0.63) in men and 0.54 (range: 0.45–061) in women [18]. A more detailed description of the methods used to calculate dietary intakes and the validity of the BDHQ has been published previously [15, 16].

### Identification of dietary pattern

Dietary patterns can be assessed with different techniques: 1) hypothesis-driven (a priori) techniques using score-based approaches such as the Mediterranean diet score or healthy eating index, 2) data-driven (a posteriori) techniques using statistical methods such as factor analysis/principal component analysis (PCA) and cluster analysis, and more recently 3) reduced rank regression (RRR), which combines characteristics of the former two approaches [20]. In contrast to a priori approaches and RRR, a posteriori approaches are data-driven, and thus, do not require a prior knowledge or theory of the diet-disease relationship and biological pathways [2]. Factor analysis is especially useful for exploring underlying patterns that explain variation in how subjects eat, and generates ranked scores, which have excellent characteristics for use in linear regression analysis, with good power for detecting associations with outcome variables [20]. We therefore chose factor analysis to identify dietary patterns in the SONIC Study. Before the analysis, individual food items calculated from the BDHQ (58 foods) were classified into one of the 33 predefined food groups (see Additional file 1: Table S1). The food-grouping scheme was generally based on the principles of similarity of nutrient profiles or culinary usage of the foods, mainly according to the food composition tables of Japanese foods [19], the classification of food groups used by the National Nutrition Survey [13], and that used in previous studies [21, 22]. Intakes of these food groups (g food/d) were adjusted for total energy intake by the density methods (g/1000 kcal). Factor analysis was conducted using the FACTOR procedure in SAS statistical software package version 9.4 (SAS Institute, Inc., Cary, NC, USA). The factors were rotated by orthogonal transformation (Varimax rotation function in SAS) to achieve a simpler structure with greater interpretability. The number of factors that best represent the data is chosen on the basis of eigenvalues, scree plots, and the interpretability of food combinations on each factor. We repeated the same analysis with a varied numbers of factors and randomly divided the subjects into two groups to examine whether these choices affected the reliability of our findings. The results showed closely similar dietary patterns (data not shown). The proportion of variance explained by each factor was calculated by dividing the sum of the squares of the respective factor loadings by the number of variables. The factor scores for each pattern and for each individual were determined by summing the intake of each food group weighted by the factor loading [23]. We used these scores to rank the subjects according to the degree to which they conformed to the dietary pattern.

### Assessment of cognitive function

Cognitive and functional status was assessed using the Japanese version of the Montreal Cognitive Assessment (MoCA-J) by trained geriatricians and psychologists [24]. The MoCA is a brief cognitive screening tool for detecting mild cognitive impairment in older people, and assesses nine domains of cognition: 1) attention, 2) concentration, 3) executive functions, 4) memory, 5) language, 6) visuoconstructional skills, 7) conceptual thinking, 8) calculations, and 9) orientation [24, 25]. The total MoCA score ranges from 0 to 30, with higher scores reflecting higher cognitive function. The test has been broadly used in other large-scale studies on cognitive function, and the reliability and validity of the MoCA for predicting early cognitive decline in comparison with a conventional cognitive test has been well established in the Japanese population [24]. We therefore used the MoCA-J total score as an indicator of cognitive function.

### Assessment of confounding factors

We collected information on sex, date of birth, residential area (Itami, Asago, Itabashi, or Nishitama), education [<10 (junior high school or less), 10–12 (high school), or ≥13 years (college or higher)], frequency of going outdoors (<1, 1–4, or ≥5 times per week), current smoking status (yes or no), alcohol consumption (yes or no), and medical history of chronic diseases (e.g., hypertension, diabetes, cancer, coronary heart disease, or cerebrovascular disease) through self-administered questionnaires at baseline. At the medical and physical examinations which were also conducted as part of the baseline survey, height and weight were measured to the nearest 0.1 cm and 0.1 kg, respectively, in light clothing without shoes. BMI (kg/m^2^) was calculated as weight (kg) divided by height squared (m^2^). Blood pressure in the left and right arm was measured twice by a physician or trained nurse using a standard mercury sphygmomanometer or electronic monitor, and the mean value of the two measurements in each of the two arms was used. Hypertension was defined as systolic blood pressure ≥ 140 mmHg or diastolic blood pressure ≥ 90 mmHg, or as having received antihypertensive treatment [26]. Venous blood samples for measurement of plasma glucose, plasma lipids, and other biochemistry indicators were collected in the fasting status. Diabetes was defined as a fasting plasma glucose concentration ≥ 7.0 mmol/L (126 mg/dL), a casual plasma glucose concentration ≥ 11.1 mmol/L (200 mg/dL), HbA1c ≥6.5%, or current use of medication for diabetes [27]. ApoE gene polymorphic alleles are the main genetic determinants of Alzheimer’s disease (AD) risk: the ε4 allele is most highly associated with AD at a large range of ages and in all ethnic groups [28]. We therefore analyzed the ApoE gene polymorphisms, and individual carriers of at least one ε4 allele (sum of ε4/ε4, ε4/ε3 and ε4/ε2) were considered to have a positive genotype. Genomic DNA was extracted from white blood cells and polymerase chain reaction methods were performed for genotyping of ApoE-ε4 allele. Depressive symptoms were assessed according to the five-item short form of the Geriatric Depression Scale (GDS-5: score range from 0 to 5) [29].

### Statistical analysis

Descriptive data are presented as means (standard deviation or 95% confidence interval) for continuous variables and percentages of subjects for categorical variables. To avoid biased grouping due to variation in body size and energy requirement, values of nutrient and food intake were energy-adjusted using the density method (i.e., percentage of energy for energy-providing nutrients and amount per 1000 kcal of energy for other nutrients and foods). The factor scores for each dietary pattern were categorized into quartiles which were used for the comparison of nutrient intakes and other lifestyle factors. Pearson correlation coefficients were used to describe the relations of nutrient intakes with each dietary pattern.

Univariate and multiple linear regression analyses were performed to explore associations of each dietary pattern with MoCA-J score. We calculated mean (95% CI) values of the MoCA-J score according to the quartile of each dietary pattern with or without adjustment for the following potential confounders: sex (men or women), area of residence (Western-urban, Western-rural, Eastern-urban, or Eastern-rural), education (<10, 10–12, or ≥13 years), frequency of going outdoors (<1, 1–4, or ≥5 times per week), current smoking status (yes or no), alcohol consumption (yes or no), BMI (kg/m^2^, continuous), GDS-5 depression score (continuous), hypertension (yes or no), diabetes (yes or no), medical history of cerebrovascular disease (yes or no), and ApoE-ε4 allele carrier status (yes or no). We also calculated unstandardized partial regression coefficient (95% CI) that reflects the change in MoCA-J score per one-quartile increase in conformity to a particular dietary pattern. Tests for trend associations were based on assigning ordinal numbers 0–3 to the four categories of each dietary pattern. All statistical analyses were performed using the SAS statistical software package version 9.4 (SAS Institute, Inc., Cary, NC, USA). All reported *P* values are two-tailed and *P* < 0.05 was considered to be statistically significant.

## Results

### Dietary patterns

Three dietary patterns were identified (Table 1) and each was labelled according to the food groups with high loadings. Factor 1, which loaded heavily on green and dark yellow vegetables, other vegetables, soy products, seaweeds, mushrooms, potatoes, fruit, salad vegetables, fish, green tea, and pickled vegetables, was labelled the ‘Plant foods and fish’ pattern. Factor 2, with high positive loadings for rice and miso soup, and high negative loadings for bread, fats and oils, and ice cream, was labelled the “Rice and miso soup” pattern. Factor 3, with high loadings for seasonings, shellfish, chicken, red meats, fish, seafood, and processed meats, was labelled the ‘Animal food’ pattern. Overall, the three dietary patterns accounted for 24.9% of the variance in food intake.

**Table 1:** Factor-loading matrix for the major three dietary patterns identified among 635 Japanese men and women aged 69–71 years who participated in the SONIC Study, Japan^a^

	Factor 1	Factor 2	Factor 3
	Plant foods and fish	Rice and miso soup	Animal foods
Other vegetables	0.71	−0.03	0.11
Green and dark yellow vegetables	0.68	−0.26	0.13
Soy products	0.55	0.22	0.06
Seaweeds	0.54	0.11	0.12
Mushrooms	0.53	−0.07	0.22
Potatoes	0.51	0.20	−0.10
Fruit	0.48	−0.22	−0.16
Salad vegetables	0.41	−0.26	0.18
Green tea	0.33	0.18	−0.04
Pickled vegetables	0.33	0.07	−0.04
Low-fat milk	0.04	−0.01	0.02
Soft drinks	−0.31	−0.21	−0.04
Alcoholic beverages	−0.32	0.04	0.07
Cooked rice	−0.33	0.74	−0.21
Miso soup	0.00	0.53	0.03
Fruit and vegetable juice	0.05	−0.20	0.12
Coffee	−0.09	−0.23	0.00
High-fat milk	0.24	−0.27	−0.08
Noodles	−0.24	−0.29	0.01
Black and Oolong tea	0.13	−0.30	−0.05
Processed meats	−0.10	−0.34	0.30
Ice cream	−0.17	−0.40	−0.11
Fats and oils	−0.05	−0.48	0.33
Breads	−0.16	−0.53	−0.14
Seasonings	0.18	0.12	0.73
Shellfish	−0.02	−0.02	0.44
Chicken	0.06	−0.15	0.43
Red meats	0.02	−0.07	0.42
Fish	0.38	0.14	0.41
Seafood	0.11	0.13	0.40
Sugar	−0.04	−0.02	0.22
Eggs	0.15	−0.12	0.19
Confectioneries	0.16	−0.22	−0.52
Percentage of variance	10.6%	7.5%	6.8%

^a^Absolute values <−0.25 or >0.25 are underlined

### Associations of dietary patterns with subject characteristics

Characteristics of the 635 analytical subjects are shown in Table 2. Compared with other participants in the baseline survey of the 70s SONIC cohort (*n* = 365), those included in the analysis were more likely to live in a rural area (*P* < 0.01) but less likely to have diabetes (*P* < 0.001). There was no difference in sex, education, frequency of going outdoors, smoking, alcohol consumption, energy intake, BMI, GDS-5 score, hypertension, diabetes, cerebrovascular disease, or ApoE-ε4 allele carrier status between the subjects analyzed and the remaining participants.

**Table 2:** Characteristics of 635 subjects aged 69–71 years who participated in the SONIC Study, Japan^a^

	Total (*n* = 635)	Factor 1: Plant foods and fish			Factor 2: Rice and miso soup			Factor 3: Animal foods		
		Q1 (*n* = 158)	Q4 (*n* = 159)	*P* value^b^	Q1 (*n* = 158)	Q4 (*n* = 159)	*P* value^b^	Q1 (*n* = 158)	Q4 (*n* = 159)	*P* value^b^
Sex (%)										
Men	46.0	72.8	23.9	<0.001	38.6	56.6	0.002	41.1	43.4	0.34
Women	54.0	27.2	76.1		61.4	43.4		58.9	56.6	
Area of residence (%)										
Western-urban (Itami)	20.0	32.3	11.3	0.004	38.0	10.1	<0.001	21.5	21.4	0.22
Western-rural (Asago)	26.0	23.4	32.1		17.1	40.3		36.1	22.6	
Eastern-urban (Itabashi)	25.4	20.9	28.9		27.2	16.4		15.8	29.6	
Eastern-rural (Nishitama)	28.7	23.4	27.7		17.7	33.3		26.6	26.4	
Education (years)										
< 10	25.5	34.2	18.2	<0.001	17.1	38.4	<0.001	22.8	23.9	0.26
10–12	47.1	44.3	47.2		50.6	36.5		46.8	53.5	
≥ 13	27.4	21.5	34.6		32.3	25.2		30.4	22.6	
Frequency of going outdoors (times per week, %)										
< 1	5.2	8.9	2.5	<0.001	2.5	7.6	0.004	5.7	5.0	0.88
1–4	29.0	34.8	25.2		24.1	31.5		31.0	32.1	
≥ 5	65.8	56.3	72.3		73.4	61.0		63.3	62.9	
Current smoker (%)	9.1	17.7	2.5	<0.001	8.2	14.5	0.03	7.6	11.3	0.22
Alcohol drinker (%)	37.0	60.1	15.1	<0.001	32.9	45.3	0.05	27.2	39.0	0.01
Energy intake (kcal/d)	1938 ± 569	1934 ± 636	1839 ± 492	0.05	1941 ± 614	1839 ± 530	0.06	2018 ± 565	1869 ± 606	0.14
BMI (kg/m^2^)	22.9 ± 3.0	23.1 ± 3.0	22.8 ± 3.1	0.58	22.9 ± 3.1	23.3 ± 3.5	0.08	22.6 ± 3.0	23.5 ± 3.7	0.02
GDS-5 score	0.88 ± 1.07	0.97 ± 1.05	0.73 ± 1.00	0.22	0.76 ± 1.01	1.02 ± 1.15	0.15	0.85 ± 1.01	1.01 ± 1.11	0.35
Hypertension (%)	65.4	70.9	62.3	0.05	63.3	66.0	0.43	59.5	71.7	0.03
Diabetes (%)	13.7	15.2	10.1	0.16	9.5	18.2	0.03	11.4	13.8	0.24
Cerebrovascular disease (%)	2.8	7.0	1.3	0.003	3.2	3.1	0.82	2.5	3.1	0.84
ApoE-ε4 allele carrier (%)	14.7	16.5	12.6	0.38	15.8	13.8	0.38	13.9	18.2	0.46

*BMI* body mass index, *GDS* Geriatric Depression Scale

^a^Values are means ± SD for continuous variables and percentages of subjects for categorical variables

^b^A linear trend test and a Mantel-Haenszel chi-square test were used for continuous and categorical variables, respectively

When the subjects were classified into four groups according to the factor score of each dietary pattern, the subjects in the highest quartile of the ‘Plant foods and fish’ pattern were more likely to be women, to live in the Western-rural area, to have higher educational attainment, and to go outdoors frequently, but they were less likely to be current smokers, to drink alcohol, or to have hypertension or cerebrovascular disease (all *P* < 0.05). The subjects with higher scores for the ‘Rice and miso soup’ pattern were more likely to be men, to be current smokers, to drink alcohol, to live in the Western-rural area, to have lower educational attainment, to go outdoors less frequently, and to have diabetes (all *P* < 0.05). The subjects with higher scores for the ‘Animal food’ pattern were more likely to drink alcohol and to have higher BMI and hypertension (all *P* < 0.05).

### Associations of dietary patterns with nutrient intakes

Mean energy-adjusted nutrient intakes for the lowest (Q1) and highest (Q4) quartiles of each dietary pattern and Pearson correlation coefficients are shown in Table 3. Across the distribution of each dietary pattern, there were differences in patterns of nutrient intake. The Pearson correlation coefficients between each dietary pattern and intake of nutrients that may be protective against cognitive impairment, namely, fatty acids, antioxidants (vitamins A, E, and C), and B-vitamins (vitamins B_6_, B_12_, and folate), were examined. The ‘Plant foods and fish’ pattern was positively correlated with intake of protein, fat, polyunsaturated fatty acids (PUFA), eicosapentaenoic acid (20:5 n-3) (EPA) + docosahexaenoic acid (22:6 n-3) (DHA), dietary fiber, and all vitamins and minerals examined. The ‘Rice and miso soup’ pattern was positively correlated with intake of carbohydrate, but negatively correlated with intake of fat, saturated fatty acids (SFA), monounsaturated fatty acids (MUFA), PUFA, and vitamins A, E, and C. In contrast, the ‘Animal food’ pattern was positively correlated with intake of protein, fat, MUFA, PUFA, EPA + DHA, vitamins A, E, B_6_, and B_12_, and all minerals examined, but negatively correlated with intake of carbohydrate.

**Table 3:** Nutrient intakes for the lowest (Q1) and highest (Q4) quartiles of three dietary patterns identified for 635 subjects aged 69–71 years who participated in the SONIC Study, Japan

Nutrient^a^	Factor 1: Plant foods and fish				Factor 2: Rice and miso soup				Factor 3: Animal foods			
	Q1 (*n* = 158)	Q4 (*n* = 159)	*P*_trend_^b^	Pearson *r* ^c^	Q1 (*n* = 158)	Q4 (*n* = 159)	*P*_trend_^b^	Pearson *r* ^c^	Q1 (*n* = 158)	Q4 (*n* = 159)	*P*_trend_^b^	Pearson *r* ^c^
Protein (% of energy)	13.7 ± 2.6	18.5 ± 3.0	<0.001	0.55	16.2 ± 2.9	16.7 ± 2.5	0.92	0.01	14.3 ± 2.4	19.1 ± 3.3	<0.001	0.60
Fat (% of energy)	22.6 ± 5.6	26.6 ± 4.8	<0.001	0.26	28.8 ± 4.7	25.2 ± 4.8	<0.001	−0.58	22.6 ± 4.5	28.5 ± 5.2	<0.001	0.42
SFA (g/1000 kcal)	6.6 ± 2.0	7.6 ± 1.6	<0.001	0.19	8.8 ± 2.0	7.1 ± 1.9	<0.001	−0.63	7.1 ± 2.0	7.9 ± 1.9	<0.001	0.16
MUFA (g/1000 kcal)	9.0 ± 2.4	10 ± 2.2	<0.001	0.15	11.4 ± 2.1	9.8 ± 2.0	<0.001	−0.59	8.5 ± 1.8	11.3 ± 2.3	<0.001	0.45
PUFA (g/1000 kcal)	6.2 ± 1.6	7.4 ± 1.4	<0.001	0.29	7.4 ± 1.4	7.1 ± 1.2	<0.001	−0.35	5.9 ± 1.2	7.9 ± 1.5	<0.001	0.51
EPA + DHA (g/1000 kcal)	0.47 ± 0.29	0.76 ± 0.37	<0.001	0.32	0.56 ± 0.27	0.68 ± 0.40	<0.001	0.15	0.44 ± 0.23	0.88 ± 0.38	<0.001	0.52
Carbohydrate (% of energy)	54.5 ± 9	53.5 ± 7.1	0.53	−0.06	51.5 ± 7.1	52.2 ± 5.9	<0.001	0.24	60.0 ± 5.2	47.0 ± 5.9	<0.001	−0.66
Dietary fibre (g/1000 kcal)	5.5 ± 1.2	10.3 ± 2.0	<0.001	0.89	8.1 ± 2.3	7.6 ± 2.0	<0.001	−0.19	7.8 ± 2.5	7.7 ± 2.1	0.60	−0.01
Vitamin A (μg/1000 kcal)	348 ± 175	599 ± 184	<0.001	0.48	520 ± 220	479 ± 217	<0.001	−0.20	444 ± 198	558 ± 258	<0.001	0.22
Vitamin E (mg/1000 kcal)	3.4 ± 0.9	5.2 ± 1.0	<0.001	0.67	4.9 ± 1.0	4.4 ± 0.9	<0.001	−0.48	3.9 ± 1.0	4.9 ± 1.0	<0.001	0.40
Vitamin B_6_ (mg/1000 kcal)	0.6 ± 0.1	1.0 ± 0.2	<0.001	0.77	0.8 ± 0.2	0.8 ± 0.2	0.42	−0.06	0.7 ± 0.2	0.9 ± 0.2	<0.001	0.46
Vitamin B_12_ (mg/1000 kcal)	5.2 ± 2.7	7.3 ± 3.2	<0.001	0.24	6.0 ± 2.5	7.1 ± 2.3	0.003	0.13	4.5 ± 1.8	9.3 ± 3.5	<0.001	0.62
Folate (μg/1000 kcal)	159 ± 38	311 ± 67	<0.001	0.85	240 ± 73	228 ± 62	0.001	−0.16	221 ± 75	247 ± 75	0.002	0.16
Vitamin C (mg/1000 kcal)	52 ± 17	118 ± 31	<0.001	0.81	90 ± 33	81 ± 31	<0.001	−0.22	83 ± 33	86 ± 33	0.31	0.06
Sodium (mg/1000 kcal)	2324 ± 495	2671 ± 542	<0.001	0.27	2541 ± 450	2615 ± 408	0.94	0.02	2178 ± 412	2914 ± 452	<0.001	0.58
Potassium (mg/1000 kcal)	1159 ± 231	2082 ± 364	<0.001	0.88	1692 ± 417	1576 ± 372	<0.001	−0.20	1516 ± 430	1742 ± 436	<0.001	0.22
Calcium (mg/1000 kcal)	257 ± 79	448.8 ± 104	<0.001	0.67	374 ± 109	346 ± 101	<0.001	−0.16	335 ± 103	394 ± 131	<0.001	0.20
Magnesium (mg/1000 kcal)	123 ± 20	192 ± 294	<0.001	0.83	157 ± 31	157 ± 29	0.36	−0.05	145 ± 33	171 ± 36	<0.001	0.30
Iron (mg/1000 kcal)	3.7 ± 0.7	6.2 ± 1.0	<0.001	0.81	4.9 ± 1.1	5.0 ± 1.0	0.59	−0.05	4.6 ± 1.2	5.4 ± 1.2	<0.001	0.31

*SFA* saturated fatty acids, *MUFA* monounsaturated fatty acids, *PUFA* polyunsaturated fatty acids, *EPA* eicosapentaenoic acid, *DHA* docosahexaenoic acid

^a^All nutrients were energy-adjusted using the density method. Means ± SD (all such values)

^b^A linear trend test was used

^c^Pearson correlation coefficient of >0.2 or <−0.2 was considered significant

### Associations of dietary patterns with cognitive function

The associations of each dietary pattern with MoCA-J score as an indicator of cognitive function are shown in Table 4. There was a strong positive association between the ‘Plant foods and fish’ pattern and MoCA-J score (*P* < 0.001). This association remained after adjustment for potential confounding effects of sex, area of residence, education, frequency of going outdoors, current smoking status, alcohol consumption, BMI, ApoE-ε4 allele carrier status, and medical history of diabetes, hypertension, cerebrovascular disease, and depression (*P* < 0.001). The ‘Rice and miso soup’ pattern was significantly associated with a lower MoCA-J score (*P* < 0.001), but this negative association disappeared after adjustment for confounders (*P* = 0.33). In contrast, no associations were observed between the ‘Animal food’ pattern and MoCA-J score.

**Table 4:** Association between dietary pattern and MoCA-J score among 635 Japanese men and women aged 69–71 years who participated in SONIC Study, Japan

		MoCA-J score^a^			
		Crude		Adjusted^b^	
	*n*	Mean	95% CI	Mean	95% CI
Factor 1: Plant foods and fish					
Q1	158	22.0	(21.5, 22.5)	22.2	(21.7, 22.7)
Q2	159	22.6	(22.1, 23.2)	22.7	(22.2, 23.2)
Q3	159	22.6	(22.1, 23.1)	22.5	(22.0, 23.0)
Q4	159	23.9	(23.4, 24.4)	23.7	(23.2, 24.2)
Effects per one-quartile increase^c^		0.56	(0.33, 0.79)	0.41	(0.17, 0.65)
*P* for trend		<0.001		<0.001	
Factor 2: Rice and miso soup					
Q1	158	23.4	(22.9, 24.0)	23.0	(22.5, 23.5)
Q2	159	23.0	(22.5, 23.5)	22.9	(22.4, 23.4)
Q3	159	22.5	(21.9, 23.0)	22.5	(22.0, 23.0)
Q4	159	22.2	(21.7, 22.7)	22.7	(22.2, 23.2)
Effects per one-quartile increase^c^		−0.42	(−0.65, −0.19)	−0.16	(−0.38, 0.07)
*P* for trend		<0.001		0.33	
Factor 3: Animal foods					
Q1	158	22.8	(22.3, 23.3)	22.6	(22.1, 23.1)
Q2	159	22.5	(22.0, 23.0)	22.5	(22.0, 23.0)
Q3	159	23.0	(22.5, 23.6)	23.1	(22.7, 23.6)
Q4	159	22.7	(22.2, 23.2)	22.8	(22.4, 23.3)
Effects per one-quartile increase^c^		0.01	(−0.22, 0.25)	0.13	(−0.09, 0.35)
*P* for trend		0.95		0.44	

MoCA-J: Japanese version of the Montreal Cognitive Assessment

^a^All values are mean and 95% CIs in parentheses

^b^Adjusted for sex (men or women), residential area (Western-urban, Western-rural, Eastern-urban, or Eastern-rural), education (<10, 10–12, or ≥13 years), frequency of going outdoors (<1, 1–4, or ≥5 times per week), current smoking status (yes or no), alcohol consumption (yes or no), BMI (kg/m^2^, continuous), GDS-5 score as depression (continuous), hypertension (yes or no), diabetes (yes or no), cerebrovascular disease (yes or no) and ApoE-ε4 allele carrier (yes or no)

^c^Values are unstandardized partial regression coefficients and 95% CIs in parentheses indicating the change per one-quartile increase in conformity to dietary pattern

In further analyses, we conducted same analyses by examining dietary patterns for men and women separately because eating behavior may differ according to sex. The three similar dietary patterns were observed in both sexes, and the results indicating a positive association between the ‘Plant foods and fish’ pattern and MoCA-J score did not change materially (data not shown). Because our findings came from a cross-sectional study, we could not exclude the possibility of reverse causation such that the subjects changed their dietary behavior or food choices because of the worsening of their cognitive function. We therefore checked the robustness of our findings through additional analysis. We ran the models while excluding 186 subjects who reported substantial changes in diet for any reason, but the results (Model 2) did not change materially [MoCA-J score per one-quartile increase in conformity to dietary pattern: β = 0.37 (95% CI: 0.08, 0.65), *P* for trend = 0.01 for the ‘Plant foods and fish’ pattern, β = −0.07 (−0.34, 0.20), *P* for trend = 0.99 for the ‘Rice and miso soup’ pattern, and β = 0.15 (−0.12, 0.41), *P* for trend = 0.33 for the ‘Animal food’ pattern].

## Discussion

The principal finding of this cross-sectional study was that the ‘Plant foods and fish’ dietary pattern was associated with better cognitive function in older Japanese men and women, as indicated by a higher MoCA-J score. These associations persisted after adjustment for potential confounding factors including sociodemographic and lifestyle characteristics, medical conditions, and ApoE-ε4 allele. In contrast, neither the ‘Rice and miso soup’ nor ‘Animal food’ patterns was associated with cognitive function. Although our results require replication, these findings provide further evidence to suggest that a healthy diet with a variety of food groups could play a protective role in slowing age-related cognitive decline in older non-Western populations.

There is now a growing body of evidence from Western populations, mostly from the United States and the Mediterranean countries of Europe, showing that healthy dietary patterns could have a protective role against cognitive impairment and the risk of dementia [2–7]. Much attention has been paid to the effect of the Mediterranean dietary pattern, characterized by high consumption of fruits, vegetables, legumes, cereals, fish, and olive oil and low consumption of meat and dairy products with moderate consumption of wine, and most studies have found an inverse association between adherence to the Mediterranean diet and cognitive impairment and dementia [3–5, 7]. In the present study, we identified the ‘Plant foods and fish’ dietary pattern as a healthy diet, which has some similar characteristics with the Mediterranean diet observed in the Western populations. In addition, our finding of better cognitive function among older people who have a higher score in the ‘Plant foods and fish’ dietary pattern is generally in agreement with those of the previous Western studies [2–7], notwithstanding the differences in study population, history or cultural influence on eating habits, dietary assessment methods used, and approaches to dietary pattern identification. The protective effect of healthy dietary patterns on cognitive function was also supported by limited studies of Asian countries [8, 9, 12], but not of others [10, 11]. In a cross-sectional study of Chinese men and women aged >65 years in Hong Kong [8], a ‘vegetables-and-fruits’ pattern (rich in vegetables, fruits, soy and soy products, and legumes) identified by factor analysis was associated with a reduced risk of cognitive impairment in women, but not in men. In a prospective study of older Japanese people aged 60–79 years in the Hisayama Study [9], a dietary pattern with high intakes of soybean and soy products, green vegetables, other vegetables, algae, and milk and other dairy products and lower intake of rice, which the authors of that study identified by means of RRR, is associated with reduced risks of all-cause dementia, AD, and vascular dementia after 15 years of follow-up. The analysis from a recent study of the Ohsaki Cohort Study has also provided further evidence that a Japanese dietary pattern derived by PCA was associated with a decreased risk of incident dementia after 5.7 years [12]. Our results, together with growing evidence from previous studies, suggest that a dietary pattern with a greater intake of vegetables, soy products, seaweed, fruits, and fish and a low intake of rice, soft drinks, and alcoholic beverages has the significant potential to promote better preservation of cognitive function in older Japanese people.

The mechanisms that underlie the associations between dietary patterns and cognitive function are not fully known, but antioxidants, which are key components of a healthy dietary pattern, may be involved [30]. Oxidative stress increases with aging; this process, together with the diminishment of the antioxidant defence system, has been indicated in the pathophysiological process of cognitive impairment and dementia [30–32]. Antioxidants such as vitamins A, E, and C in the diet may protect against neurodegeneration by scavenging free radicals [33, 34]. The beneficial effect of antioxidants from the diet might therefore be more important for older people whose antioxidant demands are high. Concerning other potential dietary factors, the favorable effects of marine-origin n-3 PUFA including EPA and DHA on cognitive decline and/or dementia have been well established in several epidemiologic studies [35–37]. This effect could be related to the role of fatty acids in maintaining the structural integrity of neuronal membranes and determining the fluidity of synaptosomal membranes and thereby regulating neuronal transmission [38, 39]. In addition, the protective effect of PUFA could be related to the anti-inflammatory effects and vascular protection exercised by these fatty acids [38, 39]. There is also growing evidence for the role of several B vitamins, especially folate, vitamins B_6_ and B_12_, which are required for proper DNA methylation and to prevent the accumulation of homocysteine, which potentially induce neurotoxic effects [40–42]. The association of other potential nutrients and dietary components, including fatty acids [43], vitamin D [44], and minerals (potassium, calcium, and magnesium) [45] have also examined, but results have been inconsistent. The ‘Plant foods and fish’ pattern in the present study was significantly and strongly correlated with intake of antioxidant vitamins (vitamins A, E, and C), EPA + DHA, B-vitamins (vitamins B_6_, B_12_, and folate), and minerals (potassium, calcium, iron, and magnesium). The positive association detected between the ‘Plant foods and fish’ pattern and MoCA-J score could be explained by the cumulative effects of individual nutrients which appear to correlate with the risk of dementia.

Very recent studies from Western countries have shown that dietary patterns high in red meat and processed meat were associated with poor cognitive function [46] and faster cognitive decline [47]. These findings were inconsistent with the results of the present study showing that the ‘Animal food’ pattern, characterized by high intake of meat, fish, and seasonings, was not related to cognitive function. At the nutrient level, we found that the ‘Animal food’ pattern was significantly correlated with MUFA, PUFA, EPA + DHA, B-vitamins (B_6_ and B_12_), and antioxidant vitamins, although epidemiological evidence regarding the association of these nutrients with cognitive function is controversial. The reason for the lack of association between the ‘Animal food’ pattern and cognitive function is unknown, but the effects of each individual nutrient derived from the primary food components of the ‘Animal food’ pattern (i.e., meat and fish) on cognitive function might have been offset. Further studies are needed to clarify this association.

Several limitations of the present study warrant mention. First, the study subjects were not a representative sample of older Japanese men and women in the general population, and the present findings should not necessarily be generalized. Second, the study is cross-sectional, which limits our ability to infer a causal relationship between diet and cognitive function. We cannot exclude the possibility of reverse causation (i.e., dietary change because of preceding cognitive decline or other disease). Third, we used a validated dietary questionnaire for Japanese adults [17, 18], but the validity of this method has not been examined among older people. The accuracy of this method in terms of estimating total food and nutrient intake remains a serious concern, and the results should accordingly be interpreted with caution. Furthermore, misreporting of self-reported food intake is a potential source of measurement error. However, such an error would be expected to attenuate any associations, and we do not think that misreporting on the BDHQ could explain the associations we describe. Fourth, the three major dietary patterns identified in the present study explained only 24.9% of the total variance, suggesting the existence of other dietary patterns, although the remaining patterns were less interpretable in our analysis. However, the amount of variance explained is highly comparable with other studies of older people in Asian countries that obtained dietary patterns using dietary questionnaires [8–12]. Finally, subjects with healthier food and nutrient intake profiles may be more health conscious in general and may engage in other healthy behaviours which could potentially confound associations with cognitive function. While we controlled for a large number of potential confounders, including sociodemographic and lifestyle characteristics, medical conditions, and ApoE-ε4 allele status, we cannot rule out unmeasured or residual confounding in an observational study of this kind.

In conclusion, the present cross-sectional study of a sample of older Japanese people showed that the ‘Plant foods and fish’ pattern was positively associated with cognitive function. A diet with high intake of green and other vegetables, soy products, seaweeds, mushrooms, potatoes, fruit, fish, and green tea and low intake of rice, soft drinks, and alcoholic beverages may contribute to the prevention of age-related cognitive decline. Further cross-sectional and longitudinal studies in various populations following different dietary patterns are needed to confirm these findings and to determine the importance of diet composition in slowing cognitive decline and preventing dementia in older age.

## Additional file

Additional file 1: The 33 food groupings used in dietary pattern analysis in SONIC Study, Japan. (DOC 113 kb)

## Abbreviations

AD: Alzheimer’s disease
BDHQ: Brief-type dietary history questionnaire
BMI: Body mass index
DHA: Dcosahexaenoic acid
EPA: Eicosapentaenoic acid
GDS: Geriatric Depression Scale
MoCA-J: Japanese version of the Montreal Cognitive Assessment
MUFA: Monounsaturated fatty acids
PCA: Principal component analysis
PUFA: Polyunsaturated fatty acids
RRR: Reduced rank regression
SFA: Saturated fatty acids
SONIC: Septuagenarians, Octogenarians, Nonagenarians Investigation with Centenarians
