# Supplementary material for: Association between dietary patterns and cognitive function among 70-year-old Japanese elderly: a cross-sectional analysis of the SONIC study
Source: Nutr J. 2017 Sep 11;16:56. doi: 10.1186/s12937-017-0273-2 (PMC5594454; doi:10.1186/s12937-017-0273-2)
Supplement: Additional file 1: — The 33 food groupings used in dietary pattern analysis in SONIC Study, Japan. (DOC 113 kb) [file 12937_2017_273_MOESM1_ESM.doc]

**Additional file 1** The 33 food groupings used in dietary pattern analysis in SONIC Study, Japan

| Food group | Foods in the group ^*^ |
| --- | --- |
| Cooked rice | Cooked rice (*n* = 1) |
| Noodles | Buckwheat noodles; Japanese wheat noodles; instant noodles and Chinese noodles; spaghetti and macaroni; soup consumed with noodles (*n* = 5) |
| Breads | Breads (including white bread and Japanese bread with a sweet filling) (*n* = 1) |
| Miso soup | Miso for miso soup (*n* = 1) |
| High-fat milk | Full-fat milk and yoghurt (*n* = 1) |
| Low-fat milk | Low-fat milk and yoghurt (*n* = 1) |
| Red meats | Pork and beef; Liver (*n* = 2) |
| Chicken | Chicken (*n* = 1) |
| Processed meats | Ham, sausages and bacon (*n* = 1) |
| Fish | Small fish with bones; oily fish (including sardines, mackerel, saury, amberjack, herring, eel and fatty tuna); non-oily fish (including salmon, trout, white meat fish, freshwater fish and bonito) (*n* = 3) |
| Shellfish | Squid, octopus, shrimp and clam (*n* = 1) |
| Seafood | Dried fish and salted fish; canned tuna (*n* = 2) |
| Eggs | Eggs (*n* = 1) |
| Potatoes | Potatoes (all varieties) (*n* = 1) |
| Soy products | Tofu (i.e. soya bean curd) and tofu products; natto (i.e. fermented soya beans) (*n* = 2) |
| Green and dark yellow vegetables | Carrots and pumpkins; tomatoes, tomato ketchup, boiled tomato and stewed tomato; green leafy vegetables including broccoli (*n* = 3) |
| Other vegetables | Cabbage and Chinese cabbage; radishes and turnips; other root vegetables (onions, burdock and lotus root) (*n* = 3) |
| Pickled vegetables | Salted green and yellow pickled vegetables; other salted pickled vegetables (excluding salted pickled plum) (*n* = 2) |
| Salad vegetables | Raw vegetables used in salad (cabbage and lettuce) (*n* = 1) |
| Mushrooms | Mushrooms (all varieties) (*n* =1) |
| Seaweeds | Seaweeds (all varieties) (*n* =1) |
| Fruit | Citrus fruit including oranges; strawberries, persimmons and kiwi fruit; other fruits (*n* =3) |
| Confectioneries | Rice crackers, rice cakes and Japanese-style pancakes; Japanese sweets; cakes, cookies and biscuits (*n* =3) |
| Ice cream | Ice cream (*n* =1) |
| Sugar | Sugar for coffee and black tea; sugar used during cooking (*n* = 2) |
| Fats and oils | Mayonnaise and salad dressing; oil used during cooking (*n* = 2) |
| Alcoholic beverages | Beer; sake; shochu and shochu mixed with water or a carbonated beverage; whiskey; wine (*n* = 5) |
| Green tea | Green tea (*n* = 1) |
| Black and Oolong tea | Black and oolong tea (including other Chinese tea) (*n* = 1) |
| Coffee | Coffee (*n* = 1) |
| Fruit and vegetable juice | Fruit juice and vegetable juice (100 %) (*n* = 1) |
| Soft drinks | Cola and sweetened soft drinks (including sports drinks) (*n* = 1) |
| Seasonings | Soy sauce; salt used during cooking (*n* = 2) |

^*^ These 58 food items from the diet history questionnaire were used for the identification of dietary patterns.
